# When battery exhaustion lets the lame walk: a case report on the importance of long-term stimulator monitoring in deep brain stimulation

**DOI:** 10.1186/s12883-015-0365-6

**Published:** 2015-07-19

**Authors:** Martin Sommer, Elisabeth Mirjam Stiksrud, Kajetan von Eckardstein, Veit Rohde, Walter Paulus

**Affiliations:** Department of Clinical Neurophysiology, University Medical Centre, Göttingen, Robert-Koch-Str. 40, D-37075 Göttingen, Germany; Department of Neurosurgery, University Medical Centre, Göttingen, Germany

**Keywords:** Deep brain stimulation, Subthalamic nucleus, Parkinson’s disease, Gait, Device misprogramming

## Abstract

**Background:**

Deep brain stimulation is increasingly used in the treatment of advanced Parkinson’s disease. While its short-term effectiveness is well documented, there are only few reports on long-term outcomes, and the need to repeatedly reprogram the stimulator is seldom reported.

**Case presentation:**

We present a 74-year-old man with gait impairment, which had been mistaken for worsening of the disease and only remitted when the stimulator battery was exhausted indicating that the stimulator itself had been the cause.

**Conclusion:**

This case highlights the need to repeatedly monitor not only battery capacity, but also stimulator-related side-effects for an extended period after implantation and, if necessary, to refer to centres capable of systematically reprogramming the device.

## Background

Deep brain stimulation is an effective method for reducing off times and hyperkinesia in advanced stages of idiopathic Parkinson’s disease [1, 2]. While there are controlled long-term outcome studies that confirm the on-going effectiveness of the treatment [3], the clinical results outside of controlled trials are less well documented, and subjective outcome reports are occasionally disappointing [4–6]. We present a striking example of a patient who had been wheelchair-bound for years due to stimulator-related side-effects. Once this was detected by chance because of battery exhaustion, we were able to partially improve his gait and speech clarity by reprogramming the stimulator.

## Case presentation

A 74-year-old, retired teacher with a 20-year history of idiopathic Parkinson’s disease had undergone bilateral subthalamic nucleus (STN) deep brain stimulation (DBS) in a centre with experience in the method. We first saw him seven years after the implantation when he presented in our clinic to have the device transiently switched off for scheduled dental surgery. He was wheelchair-bound outside his home and used a walking frame at home. His caregiver reported that he fell frequently, and he was dysarthric to an extent that almost completely hindered oral communication. The UPDRS III score was 32 of a possible 108 points; the DBS settings were 130 Hz with 60 microseconds pulse width and monopolar setting with the second deepest electrode contact on each side acting as cathode and the case acting as anode with an amplitude of 2.5 V in the right and 3.0 V in the left hemisphere. He was on medication with levodopa, entacapone, and pramipexole divided into seven daily doses, with a total levodopa equivalent dose of 998 mg [7], oral quetiapine 25 mg at night-time for his visual hallucinations, as well as oral rivastigmine 3 mg in the morning for dementia. The Kinetra® battery capacity was indicated as “OK” with a voltage of 2,56 V and 40–90 % of the capacity used.

Five months after our first contact, the patient’s caregiver contacted the first author and reported that the patient was surprisingly able to walk independently, and that the clarity of this speech had improved markedly. She also reported that the deep brain stimulator no longer responded to the patient’s controlling device.

We confirmed that the battery was exhausted. He was able to speak intelligibly with mild hypophonia. Rising from chair was fast, but postural responses were markedly reduced, resulting in a very unsteady, albeit independent gait over a distance of several meters. The UPDRS part III score was 24. He was now on medication with levodopa, entacapone and pramipexole every four hours, with a total levodopa equivalent dose of 1182 mg, oral quetiapine 25 mg at night-time and a rivastigmine patch 9 mg in the morning.

Expecting a relapse and worsening of the parkinsonian symptoms in the foreseeable future, we implanted an Activa PC ® device and left the stimulator off until his clinical condition should worsen. To avoid falls, we recommended that he walk only with assistance and with a walking frame.

After a further four months the patient presented with increasingly incapacitating fluctuations of motor function that made walking with the frame more and more difficult, indicating further disease progression. The other motor symptoms were stable except for a slight worsening of his hypophonia and very poor postural stability, resulting in a UPDRS part III score of 27. We carefully retested the electrodes and opted for a bipolar setting in the left hemisphere with contact 3 negative and contact 2 positive at 3.5 V, and a monopolar setting in the right hemisphere with the deepest contact 7 as cathode and the case as anode at 2.0 V, with 130 Hz frequency and 60 microseconds pulse width bilaterally. We discharged the patient with less pronounced dyskinesias, and medication with levodopa, entacapone and pramipexole in eight doses over the day, with a total levodopa equivalent dose of 1182 mg, oral quetiapine 25 mg at night-time, and oral rivastigmine 3 mg three times daily.

## Conclusion

We report on a patient with an obvious post-operative over-stimulation, which made him wheelchair-bound, which was mistaken for disease progression and undetected for years, also by ourselves at the initial presentation. An incorrectly programmed stimulator was only recognized as the cause when he was able to walk after the stimulator battery had been exhausted.

We assume that internal capsule stimulation impaired his walking. Over-stimulation causing internal capsule stimulation was a frequent finding in a recent retrospective study on causes for dissatisfaction with STN deep brain stimulation [6]. Slowly decreasing postoperative microlesional effects [8], impedances changing over months [9] or years [10] after surgery all evolve on prolonged time scales that are difficult to predict, and influence the effects and side-effects of DBS. Disease progression and the occurrence of axial and non-dopamine-responsive symptoms further complicate the clinical picture [11–14]. This extends the postoperative stimulator optimization phase to 12 months [6]. Some DBS centres advocate routinely rescheduling the patients six or twelve months after surgery [8] in order to detect secondary postoperative worsening of symptoms. Strategies to reduce axial side effects include smaller stimulation fields by bipolar settings [8], reduced stimulation frequencies reported to be effective in some patients [15–17], or interleaved stimulation of deeper electrode contacts [18].

In summary, this case exemplifies the importance of long-term postoperative stimulator programming to make the full long-term potential of deep brain stimulation available to the patient.

## Consent

Written informed consent was obtained from the patient for publication of the case report. A copy of the written consent is available for review by the Editor of this journal.
